# Accelerated Wound Healing by mTOR Activation in Genetically Defined Mouse Models

**DOI:** 10.1371/journal.pone.0010643

**Published:** 2010-05-13

**Authors:** Cristiane H. Squarize, Rogerio M. Castilho, Thomas H. Bugge, J. Silvio Gutkind

**Affiliations:** 1 Oral and Pharyngeal Cancer Branch, National Institute of Dental and Craniofacial Research, National Institutes of Health, Bethesda, Maryland, United States of America; 2 Division of Oral Pathology/Medicine/Radiology, Department of Periodontics and Oral Medicine, University of Michigan School of Dentistry, Ann Arbor, Michigan, United States of America; UCLA and Cedars-Sinai Medical Center, United States of America

## Abstract

**Background:**

The management of slow or non-healing ulcerations constitutes an increasing clinical challenge in the developed world because of the ageing of the population and the pandemic rise in type II diabetes. Recent studies suggest that molecular circuitries deployed by tumor cells to promote cancerous growth may also contribute to tissue regeneration. Here, we exploited this emerging information to search for novel molecular targets to accelerate wound healing.

**Methodology/Principal Findings:**

We found that the activation of the PI3K-Akt-mTOR pathway, whose aberrant function is a frequent event in human neoplasia, represents an integral component of the normal wound healing process. By the use of genetically defined approaches, including the epithelial-specific ablation of *Pten* and *Tsc1*, we show that mTOR activation can dramatically increase epithelial cell proliferation, migration, and cutaneous wound healing, while pharmacological inhibition of mTOR with rapamycin delays wound closure.

**Conclusions/Significance:**

Overall, our findings indicate that the transient pharmacologic activation of the PI3K-Akt-mTOR signaling axis may represent a novel clinical intervention strategy to accelerate the healing of debilitating and life-threatening wounds.

## Introduction

Humans display a remarkable capacity to heal injured tissues after external trauma to the body. This capacity, however, may be compromised by the large size of the injury, by underlying medical conditions, or by advanced age. For example, critical-size burn victims, patients who received chemotherapy and/or radiotherapy, elderly, and diabetic patients often present with slow or non-healing ulcers that constitute a significant clinical challenge, and the public health impact of non-healing wounds is likely to increase within the coming decades, due to the ageing of the world population and the pandemic rise in type II diabetes [1], [2], [3].

Certain molecular pathways that are exploited by tumor cells to promote cancer progression have been recently shown to play also a primary physiological role in the regeneration of injured tissues [4], [5], [6]. In this regard, the phosphatidylinositol 3 kinase (PI3K)-Akt pathway is one of the most frequently aberrantly activated intracellular signaling routes in cancer [7], [8], [9], [10]. The persistent activation of growth factor receptors by mutations or autocrine and paracrine mechanisms, gene amplification resulting in overexpression of PI3K and/or mutations in its coding sequence rendering this lipid kinase constitutively active, as well as decreased activity of the lipid phosphatase PTEN by multiple genetic or epigenetic events, all converge to promote the aberrant accumulation phosphatidylinositol-3,4,5-triphosphate (PIP_3_) in cancer cells, thereby causing the activation of the serine-theronine kinase Akt and its downstream targets, including the mammalian target of rapamycin (mTOR) [7], [8], [11]. These observations prompted us to explore whether the PI3K-Akt pathway participates in wound repair, as well as whether the targeted activation of this signaling pathway represents a suitable approach to accelerate wound healing. We show here that PI3K-Akt activation promotes cutaneous wound repair *via* mTor. More importantly, we demonstrate that elevating mTor activity dramatically accelerates the healing process, suggesting a novel strategy for the clinical management of slow or non-healing ulcerations.

## Results

### The Akt/mTOR signaling pathway is up regulated in wound healing

**Figure 1:**
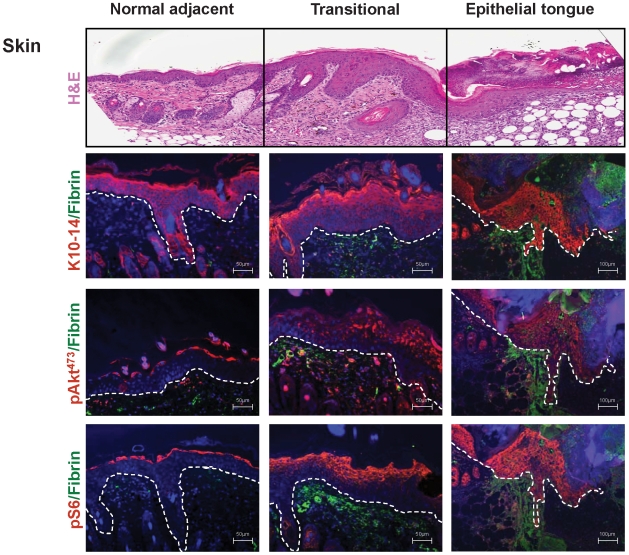
The Akt-mTOR pathway is upregulated during wound healing. (Upper panel). Representative histological sections of skin after incisional cutaneous wounds in mice. H&E stained section displaying the histological characteristics of the unwounded epithelium (normal adjacent) followed by an increase in the thickness of the spinous layer (acanthosis) (transitional), and the presence of migrating epithelial cells that form an epithelial tongue (epithelial tongue) at the wound edge. (second panel from top). Immunofluorescence for Cytokeratins 10 and 14 (K10-14, red) reveals the epidermal layer, which is delineated from the dermis by a white dotted line. Note the expansion of pAkt^473^ and pS6 (red) during wound healing from a single cell layer in the granular layer to multiple cell layers in the transitional epithelium, followed by expression in all cell layers including the basal layer in the migrating epithelial tongue (lower two panels). Cell nuclei were stained with DAPI (blue) and fibrin(ogen) with a FITC-conjugated antibody (green) in all panels. Scale bar, 50 µm and 100 µm, as indicated.

To begin addressing whether the PI3K/Akt pathway is activated during normal wound healing, we performed incisional skin wounds in mice and harvested tissues for molecular analysis. The stratified squamous epithelium adjacent to the wound site (Figure 1, normal) is organized into the basal layer that progressively differentiates into the spinous layer, granular layer and finally the stratum corneum. These four layers express distinct cytokeratins. For example, cytokeratin (K) 14 is expressed on the basal layer while and K10 is expressed in the suprabasal layers. During wound healing, migrating keratinocytes form a thin wedge-shape epithelial tongue of a few cell layers which establish the leading wound edge (figure 1- Epithelial tongue). There is also an increase in the thickness of the spinous layer adjacent to the wound and a rapid proliferation of epithelial cells immediately next to the migrating epithelial tongue (figure 1- Transitional)[1]. During the healing process, proliferation and migration are intimately related, with hyperproliferative cells in the transitional tissue feeding the migrating epithelial sheaths that form the epithelial tongue, as part of an interdependent process [1], [12].

We next analyzed the status of activation of the PI3K/Akt pathway during wound healing. In normal skin, phosphorylated forms of the S6 ribosomal protein, pS6, and serine 473 Akt, pAkt^473^, which represent downstream targets of mTOR complex 1 and mTOR complex 2 respectively [13], [14], accumulate in the granular layer, consistent with our recent findings in postnatal skin development [15]. During wound healing, however, there is an expansion of the cell layers exhibiting pAkt^473^ and pS6 in the spinous layer of the transitional epithelium, and a remarkable phosphorylation of these molecules in all layers in the migrating epithelial tongue. The most striking was the presence of pAkt^473^ and pS6 in the basal layer of the epithelial tongue, which was always absent from the normal epithelium (Figure 1).

### *Pten* excision accelerates wound closure

**Figure 2:**
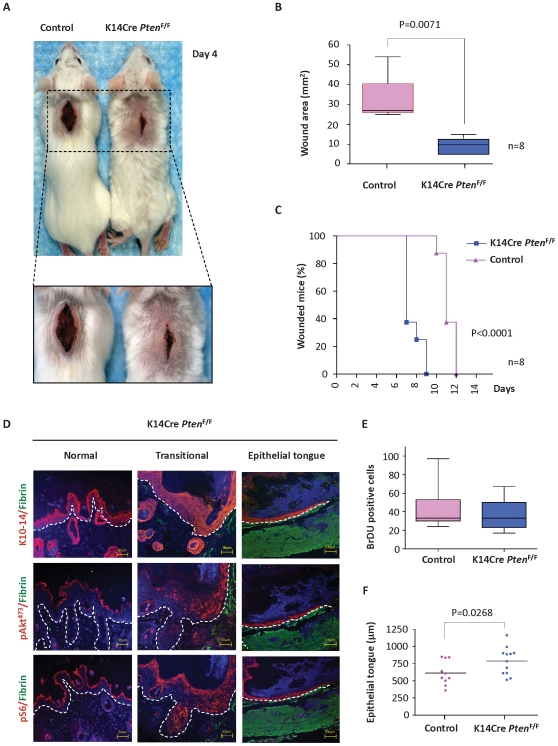
Accelerated wound closure upon epidermal *Pten* deletion: Enhanced mTor activation and re-epithelization. (A) Representative example of accelerated wound closure in *Pten-*deficient (K14Cre *Pten* ^F/F^) mice compared to control mice 4 days after skin wounding. (B) Quantification of the wound area is represented by the box-and-whiskers graphic (horizontal line, median value; *n* = 13 for each group; **p = 0.0071). (C) Percentage of mice exhibiting open wounds after surgical incision in control mice (triangles, n = 8) and K14Cre *Pten* ^F/F^ mice (squares, n = 8) at the indicated days is depicted. There was a significant increase in the rate of wound closure (***p<0.0001). (D) Representative examples of K10 and K14 (red-upper panel), pAkt^473^ (red-middle panel), and pS6 (red–lower panel) expression in K14Cre *Pten* ^F/F^ wounds as determined by immunofluorescence. DAPI (blue) and fibrin(ogen) (green). Note the accumulation of pAkt^473^ and pS6 in the transitional zone and epithelial tongue at the wound edge (transitional and epithelial tongue), when compared to the adjacent epithelium (Normal). (E) Quantification of BrDU positive cells in histological slides at the transitional and wound edge of control and K14Cre *Pten* ^F/F^ mice (*n* = 10 for each group; *p = 0.0331). (F) Quantification of epithelial migration and re-epithelization. The length of the epithelial tongue was measured in histological slides of wounded control (n = 10) and K14Cre *Pten* ^F/F^ mice (n = 12) (mean; *p = 0.0268).

To begin exploring the contribution of the PI3K/Akt/mTOR pathway to wound healing, we first perturbed this pathway by excising *Pten*, a key negative regulator of this signaling axis [11]. We specifically removed Pten from the epithelial compartment of the skin by crossing mice harboring a floxed *Pten* allele (*Pten* ^F/F^) with mice expressing the Cre recombinase under the control of the K14 promoter (K14Cre). Incisional skin wounds were performed in control and *Pten* epithelial-specific conditional knockout mice (K14Cre *Pten* ^F/F^). A clearly faster rate of healing upon *Pten* excision from the skin mice was apparent as early as 4 days after wounding (Figure 2A), which was confirmed by quantification of the wound area (p = 0.0071, Figure 2B). Analysis of wound closure also indicated that the *Pten* epithelial-specific conditional knockout mice display a significant increase in the rate of wound healing, with half of the K14Cre *Pten* ^F/F^ mice healing by day 7, compared to the control mice that displayed a median wound closure of 11 days (p<0.0001, Figure 2C).

### *Pten* excision leads to upregulation of Akt/mTor pathway and enhanced epithelial migration

**Figure 3:**
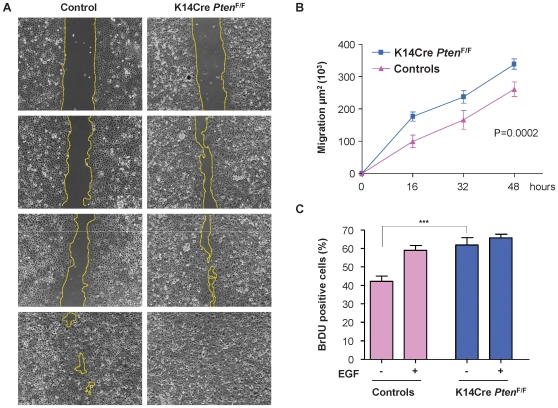
Keratinocyte migration *in vitro* is enhanced upon *Pten* excision. (A) Scratch wound assays in primary cultures of keratinocytes from control mice and K14Cre *Pten* ^F/F^ mice. Wounds were generated after cell confluence. *In vitro* cell migration and wound closure were assessed every 16 h. Areas of migration were measured in duplicates wells using keratinocytes from three distinct mice for each group (dotted line) and represented in (B) as mean ± s.e.m. (p = 0.0002) (C) Graphics shows cell proliferation represented as the percentage of BrDU incorporation by the primary keratinocytes. EGF was added where indicated (***p≤0.001).

**Figure 4:**
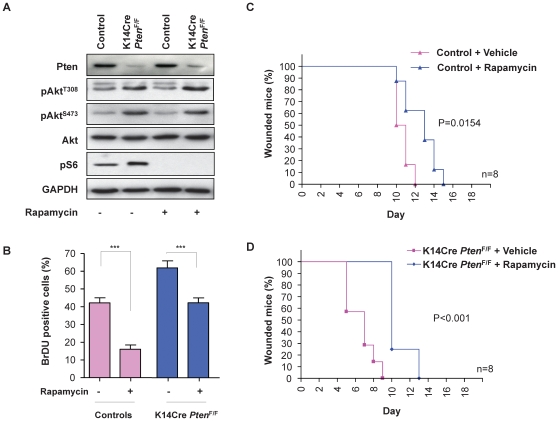
Rapamycin regulates the Akt-mTOR network in Pten-deficient keratinocytes and delays wound healing. Primary cultures of keratinocytes isolated from control and K14Cre *Pten* ^F/F^ mice were treated with vehicle or rapamycin (50 nM), as indicated. Data are representative of three independent experiments performed in triplicate. (A) Western blot analysis showing very low Pten levels in K14Cre *Pten* ^F/F^ keratinocytes, which presented increased levels of both pAkt^Thr308^ and pAkt^Ser473^. Rapamycin effectively ablates S6 phosphorylation, decreases pAkt^S473^, and increases pAkt^Thr308^ in K14Cre *Pten* ^F/F^ keratinocytes when compared to control keratinocytes. GAPDH provided loading controls. (B) Bar chart represents the percentage of BrDU positive cells in the primary culture of keratinocytes (***p≤0.001). (C and D) The graphic represents the percentage of animals with open wounds in the control mice (C) and K14Cre *Pten* ^F/F^ mice group (D) at each indicated day after cutaneous wounding. Rapamycin and vehicle administration was initiated 48 h before the wounding (see methods for details) and wound closure was used as endpoint; n = 8 for each genotype and time point; p values are indicated.

To test whether accelerated wound healing correlated with Akt and mTor activation, tissue samples were harvest at day 4 post wounding. Immunohistochemical staining of wound samples showed that the levels of pAkt^473^ and pS6 were enhanced in the suprabasal, spinous, and granular cell layers in the transitional skin of *Pten* conditional knockout mice (Figure 2D-transition) as compared to non-wounded epithelium (Figure 2D, normal). *Pten* excision enhanced epidermal proliferation as reflected by the number of BrDU-positive keratinocytes in the transitional compartment adjacent to the wound edge (Figure 2E, p = 0.0331). In addition, the wounds of K14Cre *Pten* ^F/F^ mice were characterized by the presence of an elongated migrating epithelial tongue that displayed high levels of expression of pAkt^473^ and pS6 (Figure 2D). In line with this observation, the *Pten* epithelial-specific conditional knockout mice showed accelerated re-epithelization as judged by the direct quantification of the length of the epithelial migrating tongue (p = 0.0268, Figure 2F). This increased migratory behavior could be recapitulated in primary keratinocyte cultures from control and K14Cre *Pten* ^F/F^ mice (Figure 3A–3B). As expected, Pten was almost absent in keratinocytes isolated from K14Cre *Pten* ^F/F^ mice (Figure 4A, top panel). Using a scratch wound assay, we observed that excision of *Pten* accelerated keratinocyte migration into the denuded area (p = 0.0002, Figure 3A–3B). These results are aligned with *in vitro* studies linking Pten with cell polarization and directional migration [16], [17]. In addition, the lack of Pten expression led to an enhanced proliferation of the primary keratinocytes (p<0.001, Figure 3C). The addition of EGF, a growth and pro-migratory factor that stimulates the PI3K-Akt pathway, served as an internal control. While EGF promoted the proliferation of control keratinocytes to nearly the same levels than Pten deficient cells, this growth factor failed to increase the proliferation of keratinocytes lacking *Pten*, likely because they may have already achieved a maximal proliferative capacity due to the persistent activation of Akt in the absence of Pten. Indeed, elevated levels of pAkt^308^, pAkt^473^, and pS6 were also observed in primary cultures of keratinocytes isolated from K14Cre *Pten* ^F/F^ mice (Figure 4A), albeit primary keratinocyte cultures from control mice often have demonstrable basal levels of pS6. Rapamycin treatment, which inhibits mTOR [14], [18], led to a complete ablation of pS6 phosphorylation, although it had only a limited impact on pAkt^Ser473^ levels (Figure 4A), aligned with a direct effect of rapamycin on the mTORC1 complex after short time treatment [19], [20]. Rapamycin caused a decreased proliferation of control and *Pten* knock out keratinocytes (Figure 4B), supporting a role for mTOR in epithelial cell growth in wild type cells as well as downstream from Pten.

### Rapamycin delays healing *in vivo*

Accumulated experience in the clinic suggests that prolonged rapamycin treatment may result in delayed wound healing [21], [22]. This was reflected in a significant delay in wound closure upon rapamycin treatment of control mice when compared to mice treated with vehicle (p = 0.0154, Figure 4C). We also analyzed the consequences of chronic and long term use of rapamycin by performing incisional wounds on mice treated for more than 6 months with rapamycin, which delayed wound closure to an extent similar to that caused by rapamycin administration during the acute wound healing studies (data not shown). Using this pharmacological approach, we next investigated whether mTor contributes to the acceleration of wound healing caused by Pten deletion. K14Cre *Pten* ^F/F^ mice treated with rapamycin presented a delayed wound closure when compared to vehicle injected K14Cre *Pten* ^F/F^ mice (p<0.0001, Figure 4D). These results showed that inhibition of mTOR *in vivo* abrogates the accelerated wound healing caused by *Pten* deletion in the epithelial compartment, thus suggesting an important role for mTor in the wound healing process downstream from PI3K and Pten.

### Activation of mTor in the epithelial compartment accelerates wound closure

**Figure 5:**
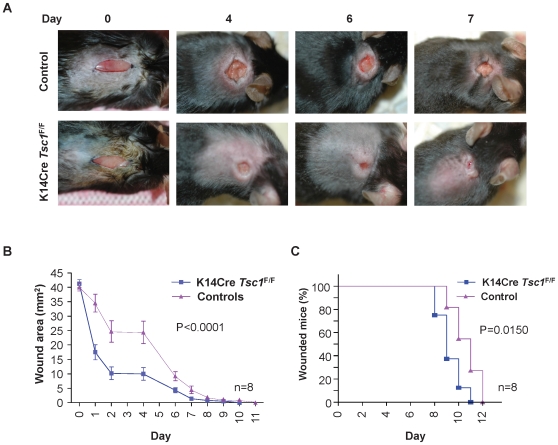
Accelerated wound closure in upon epithelial *Tsc1* excision. Representative photographs of control and K14Cre *Tsc1* ^F/F^ mice at the indicated days after wounding. (B) Quantification of the wound area. Results represent the mean ± s.e.m; n = 8 for each time point and genotype. (***p<0.0001) (C) Graphic representation of the percentage of animals displaying open wounds in control mice (A) and K14Cre *Tsc1* ^F/F^ mice (B) at each depicted time (days) (*p = 0.0150).

**Figure 6:**
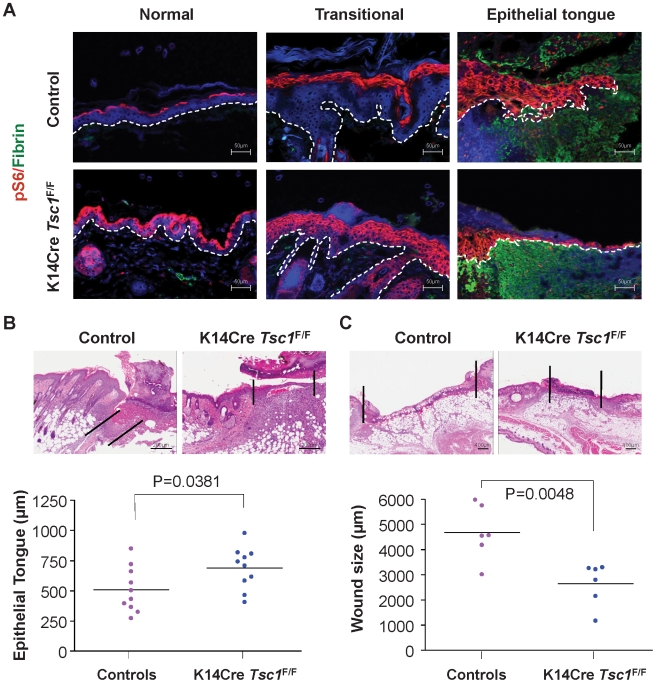
Acceleration of wound healing upon ablation of *Tsc1* in the skin. (A) Representative examples of immunofluorescence in histological sections of incisional skin wounds in mice; pS6 (red), fibrin(ogen) (green), DAPI (blue), as above. The white dotted line delineates the epithelial layer from the underlining dermis. Note the expansion of pS6 (red) expression in the normal, transitional and elongated migrating tongue of the epithelial compartment of the K14Cre *Tsc1* ^F/F^ mice when compared to control mice. Scale bar, 50 µm. (B) Representative H&E stained sections showing enhanced re-epithelization of the wound area as reflected by the length of the migrating epithelial tongue in the K14Cre *Tsc1* ^F/F^ mice when compared to the control mice. The black lines delineate the epithelial migrating tongue. Quantification of the epithelial tongue from each genotype is represented by the dot plot (n = 10 for each genotype; scale bar, 200 µm; *p = 0.0381). (C) Representative histological sections of wounded skin from control and K14Cre *Tsc1* ^F/F^ mice. The black line indicates the wound width, and the scatter plot represents the quantification of the wound width from control and K14Cre *Tsc1* ^F/F^ mice (n = 6 mice per genotype; scale bar, 400 µm; **p = 0.0048).

As rapamycin may exert multiple effects upon mTOR inhibition in the epithelial and stromal compartments of the wound area, we sought to examine the direct consequences on wound healing of the conditional activation of mTOR in the epithelial cells. The pathway linking PI3K-Akt to mTOR is initiated by phosphorylation and inactivation of the tumor-suppressor protein, tuberous sclerosis complex protein 2 (TSC2) by Akt [23]. TSC2 associates with a second tumor-suppressor protein, tuberous sclerosis complex protein 1 (TSC1), acting together as a GTPase activating protein (GAP) for the small GTPase Rheb1. The inactivation of TSC2/TSC1 complex by Akt leads to the accumulation of the GTP-bound (active) form of Rheb1, which in turn promotes the direct activation of mTOR (reviewed in [23]. Thus, as a genetically defined approach to further examine the role of epithelial mTOR in accelerated wound healing, we conditionally excised *Tsc1* from keratinocytes thereby activating mTOR by perturbing a negative regulatory step acting immediately downstream from PI3K and Akt. After wounding, the area of the wounds was measured daily (Figures 5A and 5B) and the time to wound closure was determined. Epithelial specific excision of *Tsc1* accelerates wound healing *in vivo* (figure 5A, B, & C), which is evident throughout the healing process as early as day 1 (p<0.0001, Figure 5B). Indeed, the median wound closure time in K14Cre *Tsc1* ^F/F^ mice is 9 days, as compared to day 11 in littermate control mice (p = 0.0150, Figure 5C). Histological analysis of the wounds confirmed the upregulation of mTOR signaling on the K14Cre *Tsc1* ^F/F^ mice, which displayed an overexpression of pS6 in the normal, transitional and the elongated migrating epithelial tongue of the skin epithelium when compared to the control mice (Figure 6A). Moreover, ablation of *Tsc1* in the skin compartment resulted in the increased re-epithelization in the K14Cre *Tsc1* ^F/F^ mice as judged by the length of the epithelial tongue (p = 0.0381, Figure 6B). Quantification of the wound size confirmed that K14Cre *Tsc1* ^F/F^ mice displayed accelerated wound closure (p = 0.0048, Figure 6C).

## Discussion

There is an urgent need to develop effective approaches to accelerate the healing of cutaneous wounds. In this regard, whereas tumoral growth has been often referred to as wounds that do not heal [24], we can now begin to take advantage of the emerging information on growth promoting pathways in cancer cells to accelerate the healing of injured tissues. We observed here that the activation of the PI3K-Akt-mTOR pathway, whose aberrant function is one of the most frequent events in human neoplasia, represents an integral component of the normal cutaneous healing process. Most importantly, we demonstrate that the targeted activation of this signaling pathway can dramatically accelerate wound healing rates. Indeed, by the use of pharmacological and genetically-defined approaches we found that the activation of the PI3K-Akt pathway enhances the rate of wound closure dependent on the activation of mTOR, and that the activation of mTOR in epithelial cells by genetic ablation of either of two of its upstream regulators, *Pten* and *Tsc1*, suffices to accelerate cutaneous wound healing.

An interesting observation in this study is that the activation of mTOR in normal epidermis always occurs in granular layers, which represent differentiated non-proliferative cells. This may explain why mTOR inhibitors do not exert a widespread cytostatic effect on normal epithelial cells *in vivo*, nor do they affect normal skin function, as suggested by clinical studies and our recently published reports [15], [25], [26], together suggesting that the basal keratinocytes may not strictly depend on mTOR activity to proliferate. However, during wound healing, mTOR activation is observed in the basal, subrabasal, and spinous layers of the stratified epithelium adjacent to the wound wedge and in the migrating epithelial tongue, suggesting that both the proliferative capacity and migratory activity of these cells may be controlled by mTOR. In addition, the formation of the epithelial tongue and wound closure involves of the interaction between the proliferating and migrating keratinocytes and the stromal cells. Thus, it is possible that mTOR activity in the epithelial cells may also regulate the expression of cytokines such as TGFβ, TGFα, FGF, and VEGF, which are known to contribute to the epithelial/stromal interactions during wound healing (reviewed in [27], [28], [29]). In this context, it is possible that mTOR activity in the epithelial cell compartment may facilitate wound closure by regulating epithelial cell proliferation and migration, together with its ability to regulate paracrine mechanisms involved in cellular communication processes that contribute to the wound microenvironment.

mTOR inhibitors such as rapamycin (serolimus) have been used in combination with cyclosporine or tacrolimus in the clinic since 1999 for decreasing the risk of acute allograft rejection episodes, as it helps preserve the long-term renal function better than high doses of cyclosporine (CsA) alone [26], [30]. The need to activate mTOR as part of the normal cutaneous healing process may now explain why mTOR inhibitors can cause a delay in wound closure as recently reported [21], [26]. This potential undesirable side effect could be controlled with countermeasure therapies or by the interruption of the treatment with mTOR inhibitors during the healing process [31].

On the other hand, the PI3K-Akt-mTOR pathway may be amenable for activation by chemical approaches by the use of small synthetic molecules in addition to genetically defined strategies. This could be achieved, for example, by the use of PTEN inhibitors, some of which have been recently described [32], [33]. In this regard, small molecules interfering with PTEN function can enhance wound closure and rapidly restore lung epithelial monolayer integrity following injury in relevant models *in vitro* [34], [35]. Although their current off-target toxicities may prevent their clinical application, future studies may provide opportunities for the development of therapeutically relevant agents inhibiting PTEN and other molecules on its signaling pathway as a molecular-targeted approach to accelerate wound healing upon local delivery.

Indeed, based on our current results, we can expect that the transient and localized activation of mTOR by molecules interfering with the activity of TSC1 and or TSC2 may represent a direct approach to trigger mTOR activation with limited interference with other intracellular signaling routes, thereby reducing unwanted side effects. In particular, whereas genetic defects in TSC1 and TSC2 may lead to hamartomas, a non-neoplastic proliferative disorder [36], [37], the deletion of *Tsc1* did not have a demonstrable impact on normal biology of the skin, nor did we observe spontaneous tumor formation in mice with conditional *Tsc1* deletion in the skin after more than one year of observation (not shown). These findings may provide a rationale for the future development of small molecule inhibitors of TSC1/TSC2 and/or other molecules suppressing the mTOR pathway for local delivery to accelerate wound healing. Whereas the cancer risk of this approach should be carefully examined, we have recently observed that epithelial stem cells are endowed with protective mechanisms triggering stem cell differentiation or senescence upon aberrant activation of mTOR [15]. Thus, pharmacological or genetic strategies aimed at the local activation of mTOR in the epidermis may accelerate the ability to epithelial cells to migrate and repopulate damaged skin areas hence accelerating wound closure without resulting in the aberrant growth of basal keratinocytes and their stem cells.

Overall, our present findings indicate that the transient pharmacologic activation of the PI3K/Akt/mTOR pathway may represent a novel clinical intervention strategy to accelerate the healing of critical sized, debilitating chronic, and life-threatening wounds in patients.

## Materials and Methods

### Wound healing assay and experimental mice

This study was approved by the Animal Care and User Committee (ACUC), according to NIH animal study protocols approved by the ACUC, protocol 06-408, National Institute of Dental and Craniofacial Research (NIDCR), in compliance with the “Guide for the Care and Use of Laboratory Animals”. Animals were housed on 12-h light/dark cycles and received food, standard rodent chow, and water *ad libitum* in compliance with AAALAC guidelines. The animals were observed daily by the investigators and animal care staff. Any animals displaying signs of discomfort, wasting, ruffled hair coat, hunching, or other signs indicative of distress were treated appropriately to alleviate discomfort or euthanized if recommended by animal care staff or the facility veterinary. Epithelial-specific *Pten* and *Tsc1* knockouts were obtained by crossing mice carrying the floxed *Pten* [38] and *Tsc1* alleles [39] with mice expressing Cre recombinase under the control of cytokeratin 14 promoter (*K14-Cre*) [40] as previously described [25]. Mice were anesthetized and dorsal surfaces were shaved and cleaned with 10% povidone-iodine topical solution. Fifteen millimeters full-thickness incisional skin wounds were made in the mid-dorsal area. The wound field was excised and fixed in aqueous buffered zinc formalin for 24 h, transferred to 70% ethanol, paraffin embedded and sectioned. The wound sites were monitored and measured daily utilizing wound closure as endpoint as previously described [41].

### Rapamycin administration and Bromodeoxyuridine (BrDU) incorporation *in vivo*

Freshly prepared 5-bromo-2′-deoxyuridine (BrDU) was injected intraperitoneally (i.p.) in a concentration of 100 µg/g body weight, 2 h before sacrificing the animals. Rapamycin (LC Laboratories, MA) was reconstituted in absolute ethanol at 10 mg ml^−1^ and stored at −20°C. Rapamycin was diluted in 5.2% Tween-80 (Sigma, MO) and 5.2% polyethyleneglycol (PEG-400, Hampton Research, CA), and injected intraperitoneally (i.p.) 1 mg/kg every other day, as reported [25].

### Culture of primary keratinocytes, *in vitro* wound closure (scratch) assay, BrDU incorporation, and Western blotting

Epidermis of new born mice from control and specific epidermal knockout mice were used to isolate primary murine keratinocytes as previous described [42]. Briefly, the mouse skin was peeled off, stretched out with the dermis faced down in a 60 mm culture dish, and floated on the surface of 0.25% trypsin at 4°C for 18 h. The epidermis was then separated from the dermis, minced in keratinocyte growth medium (KGM, Gibco, Carlsbad, CA) supplemented with high Ca^2+^. The undigested cornified fragments were removed by filtering the cell suspension through a sterile nylon cell strainer. The cells were collected by centrifugation, resuspended in fresh KGM Ca^2+^ supplemented with 10% FBS, and plated. After 24 h, attached keratinocytes were rinsed with PBS and incubated with fresh KGM in low Ca^2+^. Primary keratinocytes were assay with BrDU incorporation. After 24 hours starvation, cells were left untreated or treated for 24 h with 10 ng/ml EGF and 50 nM Rapamycin where indicated; and 10 µM BrDU was added for the last 2 h. Cells were fixed with Carnoy's fixative and then treated with 2 N HCl. BrDU was detected by immunofluorescence with anti-BrDU antibody (Axyll-Accurate Chemical & Scientific Corporation, Westbury, NY). Images were taken using Zeiss Axio Imager Z1 microscope equipped with an Apotome device (Carl Zeiss, Thornwood, NY). Quantitative analyses were performed by counting the total number of cells and cells expressing nuclear BrDU stain. Scratch assays were performed with keratinocytes grown to confluence on fibronectin coated wells and starved overnight. Scratches were made with a plastic pipette tip across the diameter of each well. Quantitative analysis of the scratch area of closure was performed using the Axiovision Rel. 4.7 (Carl Zeiss, Thornwood, NY). Cells were also lysed and 30 µg of cellular proteins were separated on 10% SDS-PAGE, transferred to nitrocellulose membranes, and analyzed by Western blot using primary antibodies against Akt, phospho-specific Akt (pAkt^Ser473^ and pAkt^Thr308^) and S6 (pS6) (Cell Signaling Technology, MA), PTEN (Cascade Bioscience, MA), and GAPDH (Santa Cruz Biotechnology, CA). Results are representative of three independent experiments performed in triplicate.

### Histology and Immunofluorescence

Haematoxylin-Eosin (H&E) staining was performed on formalin-fixed and paraffin-embedded 4 µm serial sections according to standard procedures. Immunofluorescence was performed on cells and tissue sections after antigen-retrieval using antibodies developed against Cytokeratin 10 (K10) and Cytokeratin 14 (K14) (Covance, Princeton, NJ), Fibrin(ogen) (Dako, CA); pAkt^Ser473^ and pS6 (Cell Signaling Technology, MA), and BrDU (Axyll-Accurate Chemical & Scientific Corporation, Westbury, NY), and rhodamine-X or FITC-conjugated secondary antibodies (Jackson Immunoresearch Laboratories, West Grove, PA) as described previously[15]. For nuclear staining, propidium iodide or mounting media with DAPI (Vector Laboratories, Burlingame, CA) was used. Images were taken using Zeiss Axio Imager Z1 microscope equipped with an Apotome device (Carl Zeiss, Thornwood, NY) and ScanScope (Aperio, CA). Quantitative analysis of the migrating epithelial tongue and wound size were done with ImageScope (Aperio, CA) and Axiovision Rel. 4.6 (Carl Zeiss, Thornwood, NY). H&E stain and immunofluorescence were performed in all the samples from every control and transgenic mice.

### Statistical analysis

Statistical analyses were performed by ANOVA analysis of variance test, followed by the Bonferroni's multiple comparison. The Kaplan-Meier analysis followed by log rank test was performed to analyze time to wound closure. T-test was used for comparisons of histological wounded size and area, migration of the epithelial tongue, and BrDU incorporation using GraphPad Prism 4.03 (GraphPad Software, San Diego, CA). Asterisks denote statistic significance (NS, P>0.05; * P<0.05; ** P<0.01; and *** P<0.001).
